# Clinical analysis of pain after transvaginal mesh surgery in patients with pelvic organ prolapse

**DOI:** 10.1186/s12905-021-01192-w

**Published:** 2021-01-30

**Authors:** Chang Shi, Ying Zhao, Qing Hu, Runqi Gong, Yitong Yin, Zhijun Xia

**Affiliations:** Department of Gynecology and Obstetrics, Shengjing Hospital of China Medical University, No. 36, Sanhao Street, Heping District, Shenyang, 110004 Liaoning China

**Keywords:** Transvaginal mesh surgery, Mesh complications, Postoperative pain, Pelvic organ prolapse

## Abstract

**Background:**

The purpose of this study was to investigate the relevant factors of pain after transvaginal mesh (TVM) surgery for the treatment of pelvic organ prolapse and to analyse the management and relief of the pain.

**Methods:**

A multicentre retrospective study of a clinical database of patients who underwent TVM surgery was conducted, and pain related aspects were analysed.

**Results:**

A total of 1855 patients were included in the study. We divided the patients into two groups: pain-free (1805 patients) and pain (50 patients) group. The incidence of pain after TVM surgery was 2.70%, with a median occurrence time of 7.5 months. Pain mainly involved the vagina, perineum, buttocks, groin, inner thighs, and lower abdomen. Excessive intraoperative blood loss (OR = 1.284, 95% CI 0.868–2.401) and postoperative anatomic failure (OR = 1.577, 95% CI 0.952–3.104) were analysed as risk factors with statistical significance. Mesh exposure rate in the pain group was 38%, showing a significant difference between the groups (*P* < 0.01). Forty patients underwent non-surgical treatment, with a relief rate of 40.0%, 33 patients received surgical treatment, 15 underwent partial mesh removal, and 18 underwent complete mesh removal, with a relief rate of 84.8%. The total relief rate was 88% within all 50 patients suffering from pain.

**Conclusions:**

Excessive intraoperative bleeding and unsatisfactory postoperative anatomic outcomes can increase the risk of postoperative pain; mesh exposure is also associated with the pain. Most patients can get pain relief with proper management, more than half of whom may need mesh removal with differing approach.

## Background

Pelvic organ prolapse (POP) is a common pelvic floor disorder among women, with population-based epidemiologic studies reporting the prevalence range from 2.9 to 34.3% in general population [1–4]. Women have an 11–12.6% lifetime risk of surgery for POP by the age of 80 [5, 6]. Traditional surgical procedures using weak native tissues have a high risk of failure, with almost 30% of the patients requiring reoperation [7]. Transvaginal mesh (TVM) surgery, as a minimally invasive surgery, seems to provide better anatomic outcomes and appears to be an attractive option to treat POP [8, 9].

Although these devices have improved outcomes, the safety of synthetic mesh has been questioned owing to the surgical complications, prompting the US Food and Drug Administration to issue warnings about adverse events associated with the mesh [10]. Complications following TVM surgery include exposure, pain, sexual dysfunction, recurrent POP, and urogenital and rectovaginal fistulas [11]. Previous studies have reported a 1–3% incidence of pain after the pelvic floor repair procedures with mesh kits [8, 12]. As one of the main complaints and complications, pain adversely affects a patient’s quality of life to a great extent [13, 14]. The risk factors and development mechanisms of the pain have not been fully understood; relevant factors reported include patient’s overall health and oestrogen status, mesh materials, surgeon’s experience, infection, and pelvic floor muscle spasms [15–17].

The primary objective of this clinical analysis, therefore, was to identify patient and surgical factors associated with the development of pain after the TVM surgery. The secondary aim was to conduct a clinical analysis of the management and relief of the pain.

## Methods

In this multicentre retrospective study, patients who underwent TVM surgery for POP without hysterectomy were identified from the Pelvic Floor Medical Alliance of Northeast China and Inner Mongolia between January 2013 and October 2018. Patients were excluded if they had a history of chronic pain caused by endometriosis, vulvodynia, vaginismus, interstitial cystitis, or lower back conditions, since they have a higher risk of persistent postoperative pain and may not be ideal candidates for the synthetic material placement [18].

Operations were carried out by experienced urogynaecology surgeons. Relevant demographic characteristics and surgical data were extracted from patient electronic medical records. POP stage examinations were performed before and at 3 months follow-up after the surgery with the patient in a lithotomy position, according to the International Urogynecological Association (IUGA)/International Continence Society (ICS) Pelvic Organ Prolapse Quantification (POP-Q) system [19]. Objective anatomic failure was defined if any point was at stage II or beyond, according to the POP-Q, which was in accordance with previous reports [20, 21].

Patients were recommended to be followed-up at 3 months postoperatively and every 3–6 months subsequently. Follow-up included evaluating anatomic outcomes, screening for mesh exposure or erosion by physical examination, and inquiring about complications such as pain, vaginal discharge, vaginal bleeding, and sexual dysfunction. The IUGA/ICS joint terminology and classification was used to assess the postoperative complications [22]. Patients who met the criteria for category 1B-3B (provoked pain, pain during sexual intercourse, pain during physical activities, and spontaneous pain) were classified into a pain group, and the patients who did not meet the aforementioned criteria were classified into a pain-free group. For the pain group, detailed descriptions from the patients’ medical records were collected, including pain occurrence time, type, location, degree, and remission. Pain caused by an intraoperative puncture injury and surgical incision is usually relieved in a short term after surgery without intervention, and was therefore not considered a complication.

The primary treatment of choice was non-surgical, including topical oestrogen, antibiotics, 1:5000 potassium permanganate sitz bath, biofeedback therapy, or a combination of the above. Patients in whom the conservative treatment was ineffective or who had the indications for mesh removal underwent surgery. Partial mesh removal was performed in cases of limited mesh exposure with no other bothersome symptoms and when mesh contracture was found. After irrigating with saline solution, we made an incision in the exposure or contracture site, removed the involved part of the mesh, and trimmed the edges of the vaginal epithelium. When the exposures were larger, presenting with severe symptoms, or mesh arms pierced the obturator space or ischiorectal fossa, the mesh was removed as much as possible. Complete mesh removal was performed by making an incision in the vaginal epithelium, dissecting the mesh from the overlying epithelium and underlying connective tissue, and closing the vagina with an absorbable suture. After the mesh and its arms were completely removed, a concomitant prolapse repair would be performed if needed. In cases of anatomic failure with obvious pain, we partially removed the mesh and reconnected the remaining part or performed complete mesh removal and reconstructed the normal anatomic structure with other repair procedures.

### Sample size calculation

The sample size calculation was conducted by Power Analysis and Sample Size Software version 15.0 for Windows. According to statistics, 2260 patients underwent TVM surgery in a specified period. Considering problems such as incomplete data and loss of follow-up, the actual number of patients included in the study was about 80% of 2260 patients (1,808). According to earlier studies, the incidence of pain is about 2% (1–3%) [8, 12]. When the sample proportion is 0.02, a sample size of 1808 produces a two-sided 95% confidence interval (1-Alpha) with a width equal to 0.014.

### Statistical analysis

SPSS version 25.0 (IBM Statistics for Windows) was used for data analyses. Continuous variables are presented as the mean and standard deviation or median and ranges; categorical variables are summarised using number count and percentage. Independent samples t-test was used to compare continuous variables, chi-square or Fisher’s exact test was used to compare categorical variables, and Mann–Whitney U test was used to compare rank variables between the groups. For variables with statistical differences, logistic regression was used to determine the contributions of the indicators to increase the risk of pain. A *P* value < 0.05 was considered statistically significant.

## Results

A total of 2260 patients underwent TVM surgery for POP between January 2013 and October 2018. After the exclusion of 285 patients lost to follow-up, 97 with incomplete data, and 23 with baseline self-reported chronic pain, 1855 patients were included in this study. The median follow-up length with interquartile range was 24 (11, 31) months. Fifty (2.7%) patients reported postoperative pain and were classified into the pain group, and the other 1805 patients were classified into the pain-free group.

A description of baseline demographic characteristics and surgical data of both groups are shown in Table 1. All patients had menopause. The mean age was 64.97 ± 8.98 years, and patients in the pain group were older than those in the pain-free group (67.00 ± 4.85 vs 64.91 ± 9.07 years, *P* = 0.005). Differences in body mass index, parity, previous hysterectomy, previous POP surgery (without mesh implantation), and comorbidities including hypertension and diabetes mellitus were proportionally minor between the groups. Concomitant mid-urethral slings (MUS) for the treatment of stress urinary incontinence (SUI) were implanted in 244 (13.2%) patients, and no differences were noted between the two groups in concomitant MUS or surgical procedure. Moreover, patients in the pain group were likely to lose more blood during the operation (89.50 ± 17.85 vs 83.12 ± 11.05 ml, *P* = 0.015).

**Table 1:** Patients characteristics and surgical data

Characteristics	Total (n = 1855)	Pain (n = 50)	Pain-free (n = 1805)	Mean difference/OR	95% CI	*t/x*^*2*^ value	*P* value
Age, years	64.97 ± 8.98	67.00 ± 4.85	64.91 ± 9.07	2.09	0.65 to 3.53	2.91	0.005*
BMI, kg/m^2^	23.66 ± 1.79	23.98 ± 1.16	23.65 ± 1.80	0.33	− 0.01 to 0.67	1.96	0.055
Parity, n	2.27 ± 0.90	2.42 ± 0.73	2.27 ± 0.90	0.15	− 0.10 to 0.40	1.18	0.238
Hypertension	1150 (61.99)	32 (64.00)	1118 (61.94)	1.09	0.61 to 1.96	0.09	0.767
Diabetes mellitus	274 (14.77)	8 (16.00)	266 (14.74)	1.10	0.51 to 2.37	0.06	0.804
Previous hysterectomy	51 (2.75)	2 (4.00)	49 (2.71)	1.49	0.35 to 6.32	0.30	0.646
Previous POP surgery	376 (20.27)	9 (18.00)	367 (20.33)	0.86	0.41 to 1.79	0.16	0.686
Surgical procedure							
Total	1641 (88.46)	46 (92.00)	1595 (88.36)	1.51	0.54 to 4.25	0.66	0.935
Anterior	63 (3.40)	1 (2.00)	62 (3.43)	0.57	0.08 to 4.22		
Posterior	151 (8.14)	3 (6.00)	148 (8.20)	0.72	0.22 to 2.32		
Concomitant MUS surgery	244 (13.15)	7 (14.00)	237 (13.13)	1.08	0.48 to 2.42	0.03	0.858
Operation time, min	78.73 ± 11.41	81.50 ± 12.76	78.65 ± 11.37	2.85	− 0.36 to 6.06	1.74	0.082
Estimated blood lose, ml	83.28 ± 11.33	89.50 ± 17.85	83.12 ± 11.05	5.38	1.28 to 11.48	2.51	0.015*

Values are presented as mean ± SD, median (range), and n (percentage)

*BMI* body mass index, *MUS* mid-urethral slings

*P < 0.05

The POP-Q stage and values (Aa, Ba, Ap, Bp, C, D, and Tvl) of both groups before and at 3 months after the surgery are presented in Tables 2 and 3. All patients had symptoms with grades III-IV POP preoperatively, with sufficient indications for prolapse surgery deemed by their surgeons. We found no difference in preoperative POP-Q evaluation between the two groups; whereas the differences of POP-Q stage (*P* = 0.038) and values of Aa, Ba, C (*P* = 0.019, *P* = 0.029, *P* = 0.016, respectively) at 3 months postoperatively were significant. Thirty patients in the pain-free group and four patients in the pain group experienced anatomic failure with the stage II.

**Table 2:** POP-Q stage in both groups before and after surgery

Pre-operative							Post-operative (3 months)						
POP-Q stage	Pain ( n = 50)	Pain-free (n = 1805)	OR	95% CI	*x*^*2*^ value	*P* value	POP-Q stage	Pain (n = 50)	Pain-free (n = 1805)	OR	95% CI	*Z* value	*P* value
Stage 0-II	0	0	–	–	–	–	Stage 0	17 (34.00)	825 (45.70)	–	–	− 2.07	0.038*
Stage III	31 (62.00)	1168 (64.71)	–	–	0.16	0.693	Stage I	29 (58.00)	950 (52.63)	1.48	0.81 to 2.72		
Stage IV	19 (38.00)	637 (35.29)	1.12	0.63 to 2.01			Stage II	4 (8.00)	30 (1.67)	6.47	20.05 to 20.41		

Values are presented as n (percentage)

*P < 0.05

**Table 3:** POP-Q values in both groups before and after surgery

	Pre-operative						Post-operative (3 months)					
	Pain (n = 50)	Pain-free (n = 1805)	Mean difference	95% CI	*t* value	*P* value	Pain (n = 50)	Pain-free (n = 1805)	Mean difference	95% CI	*t* value	*P* value
Aa	2.08 ± 0.60	2.06 ± 0.92	0.02	− 0.16 to 0.19	0.22	0.829	− 2.41 ± 0.81	− 2.69 ± 0.47	0.28	0.05 to 0.51	2.43	0.019*
Ba	3.66 ± 1.15	3.56 ± 0.76	0.10	− 0.23 to 0.43	0.60	0.554	− 2.24 ± 1.16	− 2.61 ± 0.46	0.37	0.04 to 0.70	2.25	0.029*
C	1.90 ± 1.39	1.95 ± 1.50	− 0.05	− 0.47 to 0.37	− 0.23	0.817	− 4.83 ± 1.30	− 5.29 ± 0.67	0.46	0.09 to 0.83	2.49	0.016*
Tvl	6.58 ± 0.57	6.45 ± 0.57	0.13	− 0.03 to 0.29	0.23	0.228	6.62 ± 0.56	6.70 ± 0.54	− 0.08	− 0.23 to 0.07	− 1.04	0.300
Ap	1.46 ± 0.97	1.68 ± 0.95	− 0.22	− 0.49 to 0.05	− 1.62	0.668	− 2.73 ± 0.37	− 2.69 ± 0.35	− 0.04	− 0.13 to 0.06	− 0.75	0.456
Bp	2.26 ± 1.37	2.34 ± 1.01	− 0.08	− 0.48 to 0.31	− 0.43	0.822	− 2.62 ± 0.40	− 2.60 ± 0.31	− 0.02	− 0.13 to 0.10	− 0.33	0.745
D	1.63 ± 1.72	1.57 ± 1.27	0.06	− 0.45 to 0.56	0.23	0.108	− 6.00 ± 1.37	− 6.36 ± 0.49	0.36	− 0.04 to 0.76	1.83	0.074

Valuse are presented as mean ± SD

*P < 0.05

Logistic analysis was performed on the contribution of each indicator to the risk of pain, including age, POP-Q values, and intraoperative blood loss. The combined effects of Aa, Ba, and C points were adopted for the assessment. The results showed that patients with greater blood loss (OR = 1.28, 95% CI 0.87–2.40, *P* = 0.026) and lager POP-Q values (OR = 1.58, 95% CI 0.95–3.10, *P* = 0.011) had a higher risk of suffering from pain, while age was not statistically significant (*P* = 0.146).

**Fig. 1:**
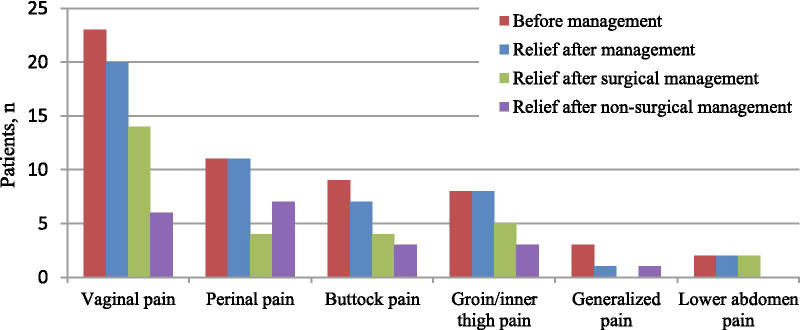
Sites of the pain and relief after treatment

The period between mesh implantation and pain occurrence ranged from 1 to 31 months, with a median of 7.5 months. The pain was localised in the vagina, perineum, buttocks, groin, lower abdomen, or multiple sites (Fig. 1). Most of the patients had spontaneous pain (88%); pain in the buttocks, groin, and inner thigh was sometimes aggravated while sitting or by certain movements such as walking, urination, and defecation. Points of tenderness or cord-like changes could sometimes be found at the physical examination in patients with vaginal pain. Mesh exposure was found in 19 patients, 13 of whom experienced vaginal discharge or developed local inflammation. The exposure rate in the pain group was 38% (19/50) and the pain-free group was 4.4% (79/1805), with a significant difference between the groups (OR = 13.39, 95% CI 7.25–24.74, *P* < 0.01).

The management measures and pain remission are summarised in Table 4 and Fig. 1. A majority of patients (80%) had received conservative treatment, including topical oestrogen, antibiotics, and physiotherapy; 33 patients, in whom the non-surgical management was unsuccessful or who had indications, received surgical intervention, including partial mesh removal for 15 patients and complete mesh removal for 18 patients. A total of 44 (88%) patients reported pain relief after the management, 16 by conservative treatment and 28 by surgical intervention.

**Table 4:** Managements for patients in pain group

Precedure	Total	Relief
Conservative treatment	40	16
Topical estrogen	32	–
Antibiotics	11	–
Physical therapy	35	–
Surgical intervention	33	28
Partial mesh removal	15	12
Mesh exposure	6	5
Mesh contracture/folding	6	6
Mesh arms piercing obturator space/ischiorectal fossa	1	0
Anatomy failure	2	1
Complete mesh removal	18	16
Mesh exposure	7	7
Mesh arms piercing obturator space/ischiorectal fossa	8	7
Anatomy failure	2	2
No abnormality on examination	1	0

## Discussion

In this retrospective study, we investigated the relevant factors of pain after TVM surgery for the treatment of POP, and analysed the management and relief of pain based on our clinical experience. The incidence of pain in our study was 2.7%. Excessive intraoperative blood loss and large postoperative POP-Q values were considered as risk factors. The vagina was the most common site of pain, followed by the perineum, buttock, and groin; few patients had lower abdomen or generalised pain. In some patients, pain could be relieved by conservative therapy. Mesh removal was an effective treatment with the relief rate of 84.8%, and mesh exposure was the most common reason for the removal.

The process by which pain develops after TVM surgery is probably multifactorial, and the manifestations of pain are varied. The relationship between excessive intraoperative blood loss and postoperative pain has not been determined. A previous study shows that excessive intraoperative blood loss can increase the risk of mesh exposure 7.3-fold [23] and may lead to postoperative inflammatory reaction, which may be related to the development of pain. One out five patients with mesh exposure in our study reported pain, compared with 0–54% in the previous studies [13, 18, 20, 24]. The exposure itself may not have been the cause of pain, while the resulting inflammatory reaction might have been relevant. It has been proposed that chronic inflammatory response caused by the exposed mesh can lead to vaginal pain [25]. Unsatisfactory postoperative anatomic outcomes may also increase the risk of pain, which may be related to the insufficient tension of the mesh. Patients with this condition may experience abdominal and perineal distension accompanied by the pain, which can also occur occasionally in untreated POP patients. In addition, although MUS is also a transvaginal implant, the concomitant MUS implantation for SUI was thought to be unrelated to the pain, in accord with previous findings [26].

Pain can sometimes resolve on its own or improve with physiotherapy, oestrogen cream, or antibiotic treatment [13, 27]. Since pain usually includes hypertonia of the pelvic floor muscles, pelvic floor physiotherapy has showed a good curative effect [28]. For the mesh exposure, more aggressive management might be required when the pain appears, rather than conservative treatment such as topical oestrogen and closure of the vaginal epithelium [27]. Partial or total mesh removal is a better option for patients who have not responded to the conservative treatment, and the rate of pain relief after the mesh removal ranges from 50 to 84% in prior studies [18].

Pain can also result from mesh arms piercing the obturator space or ischiorectal fossa [29, 30], which can be markedly improved after the mesh is removed. Thus, during the process of puncture for mesh implantation, we would like to emphasise that the implants must be placed in the interstitial space rather than in the tissue. In some cases, pain can be explained by bunching, folding, or contracture of the mesh [17, 29]. Mesh contracture may result in a concomitant contracture of the underlying pelvic floor musculature and excessive tension on the mesh arms, causing increased pelvic floor muscle tone and tenderness [17, 30]. Pain in these cases usually is unresponsive to conservative measures, but can be relieved following mesh removal. In addition, some patients had persistent pain from the moment the mesh was implanted, while no abnormality was found at the examination. In a previous study, the removal of all vaginally accessible meshes was performed in such cases [18]. Both conservative and surgical approaches in our study were used, while the improvement was unsatisfactory. The mechanism of such pain has not been clearly identified. Nevertheless, new complications may be associated with the removal of the mesh, including recurrent POP, and a concomitant prolapse repair should be performed if needed.

In this study, a small number of patients had undergone previous hysterectomy. Although several approaches including TVM for the management of POP have been reported, the best strategy for post-hysterectomy vaginal vault prolapse (VVP) remains controversial. Studies have suggested that laparoscopic sacrocolpopexy and sacrospinous fixation in the treatment of primary VVP and transvaginal bilateral sacrospinous fixation in the treatment of recurrent VVP appears to be effective and safe for the improvement of quality of life and sexual function [31]. As noted in a systematic review, TVM surgery had the highest reoperation rates (including complications and recurrence) in the treatment of VVP [32]. Therefore, we should be more careful in evaluating and selecting the appropriate approach before the TVM is intended for the reconstruction of VVP.

Health-related quality of life (HR-QoL) is widely recognised as an important outcome measure following urogynaecological surgery [33]. Taking into account the significant impact of POP on physical and mental health [34], in addition to the surgical effect, that is, anatomical reduction, surgeons should focus on the assessment of postoperative function recovery and improvement of quality of life of the patients. Therefore, multidisciplinary approach in the treatment of women with POP is very important. Although studies have shown that general HR-QoL improved significantly (mainly shown as improvement of sexual activity, mobility, excretion, depression, etc.) after apical POP reconstructive surgery [35], the use of TVM can still lead to some serious complications, such as pain, sexual dysfunction, and mesh exposure [11]. Therefore, the application of TVM should have strict indications, and is usually recommended for patients ≥ 50 years old or with low sexual activity, severe pelvic floor structural damage, or recurrence [21, 34]. Surgeons should weigh the risks and benefits on an individual level based on the patient characteristics when opting for mesh kits for surgical repair, to achieve better treatment outcomes in patients with POP.

This study had the following limitations: Firstly, pain was not directly measured with validated instruments, and the metric used for pain relief was based on subjective phrases in medical records at times. Secondly, since the mesh we used was mainly Prolift™, the factors of mesh itself (size, shape and material) are not included in the study. Then, since the pain may occur years after surgery, longer-term follow-up might be required. Finally, the cases in this study came from specific regions, and it is unclear whether the conclusions can apply to patients in other parts of the world. This study had several limitations. First, pain was not directly measured with validated instruments, and the metric used for analysis of pain relief was based on subjective phrases in medical records at times. Second, since the pain may occur years after the surgery, longer-term follow-up might be required. Third, the cases in this study were collected from specific geographical regions, and it is unclear whether the conclusions can apply to patients in other parts of the world.

Despite these limitations, the evidence of our study is strengthened by the relatively large number of patients and detailed data available from surgical and medical records. Moreover, this study investigated the relevant factors of postoperative pain, one of the inconvenient complications after the TVM surgery, the observations of which are relatively new to the available literature. The clinical practice shown in this study may provide important information about the strategy of the pain management following TVM surgery. In the future, we will carry out more in-depth studies on the mesh complications to provide more important reference for the postoperative management of TVM.

## Conclusions

The aetiology and development of pain after TVM surgery involves a variety of factors. Excessive intraoperative bleeding and failure with postoperative anatomic outcomes can increase the risk of pain, and patients with mesh exposure are more likely to develop this symptom. Patients can obtain pain relief with non-surgical or surgical management, more than half of whom need mesh removal with differing approach. More factors related to the postoperative pain remain to be explored. Through continuous research and improvement, we hope that TVM surgery can have better effects and safety.

## Data Availability

The datasets generated and/or analyzed during the current study are not publicly available due to ethical issues and potential for organizational privacy to be compromised, but are available from the corresponding author upon reasonable request.

## Abbreviations

POP: Pelvic organ prolapse
TVM: Transvaginal mesh
POP-Q: Pelvic organ prolapse quantification
SUI: Stress urinary incontinence
MUS: Mid-urethral slings
VVP: Vaginal vault prolapse
HR-QoL: Health-related quality of life
